# *ABCB1* Is Frequently Methylated in Higher-Grade Gliomas and May Serve as a Diagnostic Biomarker of More Aggressive Tumors

**DOI:** 10.3390/jcm11195655

**Published:** 2022-09-26

**Authors:** Aleksandra Majchrzak-Celińska, Arvinder Sidhu, Izabela Miechowicz, Witold Nowak, Anna-Maria Barciszewska

**Affiliations:** 1 Department of Pharmaceutical Biochemistry, Poznan University of Medical Sciences, Święcickiego 4 St., 60-781 Poznań, Poland; 2 Department of Computer Science and Statistics, Poznan University of Medical Sciences, Rokietnicka 7 St., 60-806 Poznań, Poland; 3 Molecular Biology Techniques Laboratory, Faculty of Biology, Adam Mickiewicz University, Uniwersytetu Poznańskiego 6 St., 61-614 Poznań, Poland; 4 Intraoperative Imaging Unit, Chair and Department of Neurosurgery and Neurotraumatology, Poznan University of Medical Sciences, Przybyszewskiego 49 St., 60-355 Poznań, Poland; 5 Department of Neurosurgery and Neurotraumatology, Heliodor Swiecicki Clinical Hospital, Przybyszewskiego 49 St., 60-355 Poznań, Poland

**Keywords:** *ABCB1* methylation, *IDH1* mutation, glioma, glioblastoma, diagnostic biomarker, prognostic biomarker

## Abstract

ABCB1 belongs to a superfamily of membrane transporters that use ATP hydrolysis to efflux various endogenous compounds and drugs outside the cell. Cancer cells upregulate *ABCB1* expression as an adaptive response to evade chemotherapy-mediated cell death. On the other hand, several reports highlight the role of the epigenetic regulation of *ABCB1* expression. In fact, the promoter methylation of *ABCB1* was found to be methylated in several tumor types, including gliomas, but its role as a biomarker is not fully established yet. Thus, the aim of this study was to analyze the methylation of the *ABCB1* promoter in tumor tissues from 50 glioma patients to verify its incidence and to semi-quantitively detect *ABCB1* methylation levels in order to establish its utility as a potential biomarker. The results of this study show a high interindividual variability in the *ABCB1* methylation level of the samples derived from gliomas of different grades. Additionally, a positive correlation between *ABCB1* methylation, the WHO tumor grade, and an *IDH1* wild-type status has been observed. Thus, *ABCB1* methylation can be regarded as a potential diagnostic or prognostic biomarker for glioma patients, indicating more aggressive tumors.

## 1. Introduction

Gliomas represent a diverse group of central nervous system (CNS) neoplasms with divergent prognoses. Up until 2016, gliomas were classified according to their histologic features. In 2016, the new World Health Organization (WHO) Classification of CNS tumors incorporated their molecular features alongside their immunohistology, providing a more accurate diagnosis and prognosis [1]. Due to the advances in molecular genetics last year, the WHO 2021 Classification of CNS tumors was released. This new classification encompasses the standardization of tumor grading and nomenclature and incorporates more molecular markers into CNS tumors’ classification [2,3]. Thus, the importance of the molecular diagnosis is now well understood and appreciated, and increasing efforts are being made to identify novel genetic or epigenetic biomarkers of gliomas. Such additional diagnostic or prognostic information may provide guidance for optimizing personalized therapy and improving survival rates for these often very highly aggressive tumors. For the time being, the most established prognostic factors in patients with malignant gliomas include an advanced age, an incomplete surgical resection, a glioblastoma multiforme (GBM) diagnosis, a tumor site other than the cerebrum, and a poor Karnofsky Performance Status [4]. Regarding the molecular markers, the isocitrate dehydrogenase (*IDH*) mutation and *MGMT* promoter methylation are reliable prognostic and predictive biomarkers in grade II–IV diffuse gliomas. Moreover, *TERT* promoter mutations are associated with a poorer disease course in individuals with GBM [5]. Meanwhile, novel biomarkers are being evaluated.

ABCB1, also known as MDR1 or P-glycoprotein (P-gp), is an ATP-binding cassette transporter functioning as an efflux pump, which lowers the intracellular accumulation of various anti-cancer drugs [6]. Its role in cancer has been intensively studied for decades, as its overexpression causes multidrug resistance (MDR), treatment failures, and tumor relapses [7]. The ABCB1 protein is encoded by the *ABCB1* gene, which is located on chromosome 7q21.12 and consists of 29 exons [8], with exon 1 and exon 2 being untranslated [9]. Several mechanisms have been identified to regulate the expression of *ABCB1* in cancer cells. Genomic instability, including amplifications, translocations, or gene mutations, have been reported for regions of chromosome 7, leading to increased *ABCB1* expression [10]. It is also known that polymorphisms in the *ABCB1* gene may also affect *ABCB1* expression and function [11,12,13,14]. Moreover, *ABCB1* has been demonstrated to be regulated by epigenetic mechanisms that can potentially affect inter-individual variability in treatment responses. In this regard, the *ABCB1* gene expression depends on the methylation level of the promoters used to direct its transcription. The *ABCB1* gene has two promoters, namely, downstream/proximal and upstream. In human cells, the downstream promoter, which encompasses one CpG island, along with two other CpG islands (one located in exon 1 and the other in intron 1), has been identified to be the major promoter. It is responsible for regulating most of the gene’s transcriptional activity [15]. *ABCB1* promoter methylation has been studied in breast cancer cells [10], ovarian carcinoma [16], prostate carcinoma [15], and lung adenocarcinoma [17], among others. However, *ABCB1* methylation in glioma samples remains elusive, and the potential clinical utility of its analysis is unclear to date.

Regarding glioma research, the main focus has been placed on the role of ABCB1 on chemotherapy’s efficacy [7]. It has been shown, for instance, that GBM cells overexpressing ABCB1 exhibit a high resistance to temozolomide, irinotecan, carmustine, carboplatin, and etoposide [18,19,20]. Moreover, it has been shown that the downregulation of ABCB1 enhances the efficacy of temozolomide in the GBM U87 cell line [21]. In addition, an improved brain penetration and antitumor efficacy of temozolomide have been shown as a result of ABCB1 inhibition [19]. It has also been reported that temozolomide downregulates ABCB1 expression in GBM stem cells by interfering with the Wnt3a/glycogen synthase-3 kinase/β-catenin pathway [22]. In addition, strategies of how to overcome the ABCB1-mediated MDR have been intensively investigated over the last decades. These strategies include nanocarrier technologies, antibody–drug conjugates, and ultrasound-mediated BBB opening (UMBO) [7]. Additionally, other innovative strategies including RNA interference, phytocompounds as MDR modulators with little systemic toxicity, and other physical approaches such as a combination of conventional drug administration with thermal or photodynamic strategies have also been evaluated [23].

However, the clinical role of ABCB1 in glioma patients is still under investigation; specifically, its role as a biomarker is not yet clear. Interestingly, it has been shown that the heterozygous expression of the *IDH1^R132H^* allele is sufficient to induce the genome-wide alterations in DNA methylation characteristic of gliomas [24]. Evidence exists that heterozygous *IDH1^R132H/WT^* mutations drive epigenetic instability and initiate the methylation phenotypes observed in patients [24]. Whether this aberrant DNA methylation affects the *ABCB1* promoter remains to be elucidated.

As shown in previous studies, the CpG island promoter methylation of *ABCB1* downregulates gene expression [25,26,27]. Besides serving as a potential predictive biomarker, indicating a patient’s response to treatment, *ABCB1* methylation could also be used as a diagnostic or prognostic biomarker if its levels are correlated with clinicopathological data. According to our best knowledge, such a role of *ABCB1* methylation in glioma patients has not been studied. Thus, the aim of this study was to analyze the methylation level of the *ABCB1* promoter in glioma patients of different WHO grades to establish its utility as a potential biomarker based on its relation to the *IDH1* mutation status and other clinicopathological data.

## 2. Materials and Methods

### 2.1. Glioma Samples Characteristics

This study was performed on brain glioma samples obtained from 50 patients who had undergone surgery at the Department of Neurosurgery and Neurotraumatology, Poznan University of Medical Sciences, between 2012 and 2016. Written informed consent was obtained according to protocols previously approved by the appropriate Clinical Research Ethics Committee. Surgical specimens were frozen at −80° C. The average age of patients was 50.9, while the median was 50.5. The youngest patient was 21, while the oldest was 75 years of age. Tumor samples were histologically classified by WHO’s 2007 criteria by a neuropathologist at the Department of Pathology Poznan University of Medical Sciences.

### 2.2. DNA Isolation from Tumor Samples

Genomic DNA was extracted from fresh frozen tumor samples using a commercial Genomic Mini kit (A&A Biotechnology, Gdańsk, Poland) according to the manufacturer’s instructions. DNA concentration and purity were verified using NanoDrop spectrophotometer.

### 2.3. Bisulfite Conversion and Methylation-Sensitive High-Resolution Melting (MS-HRM) of ABCB1 Promoter

Bisulfite conversion of 500 ng of genomic DNA was performed using EZ DNA Methylation Kit (Zymo Research, Irvine, CA, USA), following the manufacturer’s protocol. For the analysis of ABCB1 promoter methylation, methylation-sensitive high-resolution melting (MS-HRM) was applied. The methylation status was assessed from the melting profiles of the PCR products by comparison with melting profiles of PCR products obtained for calibration standards (methylated and unmethylated DNA, and their mixtures). As a methylated control, we used CpG-Methylated HeLa Genomic DNA (New England Biolabs, Ipswich, MA, USA), while as a negative control we employed CpGenome Universal Unmethylated DNA Set (Merck, Darmstadt, Germany). Human astrocyte genomic DNA (ScienceCell Research Laboratories, Carlsbad, CA, USA) was used as an additional negative control.

PCR was carried out in 20 µL total volume, using 5× Hot FIREPol EvaGreen HRM Mix (Solis BioDyne Co., Tartu, Estonia). Primer sequences were obtained from the literature [16]. Their sequences, annealing temperatures, and amplicon sizes are presented in Table 1. The reaction was performed according to the manufacturer’s protocol and involved 15 min. of preincubation at 95° C and 40 cycles of three-step amplification (15 s/95 °C, 20 s/Ta, 20 s/72 °C), and obtained amplicons were melted at a temperature gradient to a max of 95 °C. The obtained melting curves were normalized automatically by the calculation of the “line of the best fit” in between two normalization regions before and after the significant fluorescence decrease.

The methylation level of each sample was assessed by comparison of the PCR product normalized melting curve/peak with the normalized melting curves/peaks of the standard solutions indicating 100, 75, 50, 25, 10, and 0% methylation. However, for the samples with melting curves intersecting the methylation curves generated by the standards, a wider range of methylation levels were assessed. Eventually, using a 10% methylation threshold, we selected three groups of samples; those with methylation level above 10% were regarded as methylated, those with methylation level equal to 10% were regarded as mildly methylated, and those that matched the unmethylated control were regarded as unmethylated. All samples were analyzed in duplicate, and the reaction was repeated at least twice.

### 2.4. IDH1 Genotyping Using High-Resolution Melting Analysis (HRM)

Genotyping of *IDH1* was performed based on the assay published by Yokogami et al. in 2018 [28]. The principle of HRM-genotyping analysis is based on the discrepancies in melting curve shapes of samples with different nucleotide sequences. In the case of *IDH1^R132H^* mutation, the codon CGT changes into CAT. Thus, the wild-type samples melt at a higher temperature, while the heterozygous mutant samples melt at a lower temperature.

The reagents and the reaction protocol used for HRM analysis were identical to the one described above for MS-HRM, except that we used genomic DNA instead of bisulfite-converted DNA for the PCR amplification. Primer sequences, their annealing temperatures, and product sizes are listed in Table 1.

In order to confirm the results obtained by HRM, 12/50 (24.0%) randomly chosen PCR products were validated using bisulfite sequencing.

### 2.5. Bisulfite Sequencing

Direct HRM products for *IDH1* gene were purified using EPPiC Fast kit (A&A Biotechnology, Gdynia, Poland) and sequenced directly using the BigDye^®^ Terminator v3.1 Cycle Sequencing Kit (Applied Biosystems, Foster City, CA, USA). Cycle sequencing was performed in 20 µL reaction volume containing approx. 4 ng DNA template and 1.5 pmole sequencing primer using the recommended thermal profile and a 2720 Thermal Cycler (Applied Biosystems, Foster City, CA, USA). The HRM products were sequenced with the reverse primer. After purification of the extension products by ethanol/EDTA precipitation method or using ExTerminator kit (A&A Biotechnology, Gdynia, Poland), the DNA sequence was read by capillary electrophoresis performed in the Applied Biosystems^®^ 3130 Genetic Analyzer using the 50 cm capillary array and POP-7™ polymer (Applied Biosystems, Foster City, CA, USA).

### 2.6. Statistical Analysis

The statistical analysis was performed using Statistica 13 software (TIBCO) and PQStat v.1.8.4 (PQStat Software, Poznan, Poland). The results with *p*-value < 0.05 were considered statistically significant. The normality assumption was verified using the Shapiro–Wilk test. In order to compare differences between three independent groups, we used Kruskal–Wallis test. The relationship between categorical variables was analyzed using the Chi-Square or Fisher–Freeman–Halton test.

## 3. Results

### 3.1. Patient Sample Characteristics

The clinicopathological features, such as the patients’ age, sex, and WHO grade are summarized in Table 2. According to the WHO classification, 60.0% (30/50) of the tumor samples were grade IV, 30.0% (15/50) of the samples were grade III, and 10.0% (5/50) of the samples were grade II. There were 28 tumor samples derived from GBM patients, one gliosarcoma, five anaplastic astrocytomas, oneanaplastic, recurrent glioma, six oligoastrocytomas, two anaplastic oligoastrocytomas, two anaplastic, recurrent oligodendrogliomas, one oligodendroglioma, three fibrillary astrocytomas, and one ganglioglioma. The analyzed population consisted of 48.0% (24/50) of men and 52.0% (26/50) of women.

### 3.2. HRM Genotyping and Bisulfite Sequencing Reveal IDH1 Mutated Samples

**Figure 1:**
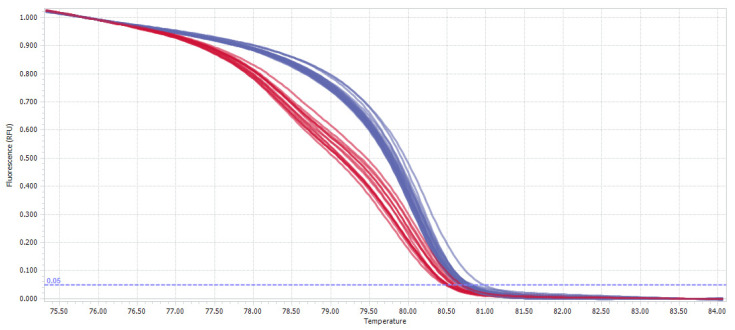
General overview of HRM results of *IDH1* mutation analysis. Representative samples derived from the *IDH1* wild-type glioma patients (melting curves colored blue), and patients with heterozygous *IDH1^R132H/WT^* mutation (melting curves colored red) are presented.

The HRM analysis allowed for the discrimination between the wild-type and *IDH1^R132H/WT^*-mutated samples (Figure 1), showing that 22.4% (11/49) of the samples were heterozygous, while 76.6% (38/49) were wild-type. For one sample, the results were not certain; thus, we excluded it from the analysis. The detailed results are presented in Table 2.

**Figure 2:**
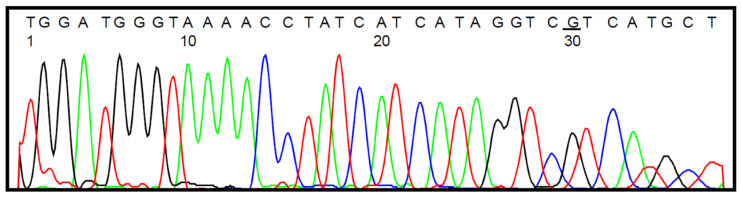
DNA sequencing data for *IDH1* target amplicon showing wild-type *IDH1* status of tumor sample #9 derived from GBM patient (the nucleotide of interest is underlined); SNP accession number: rs121913500.

**Figure 3:**
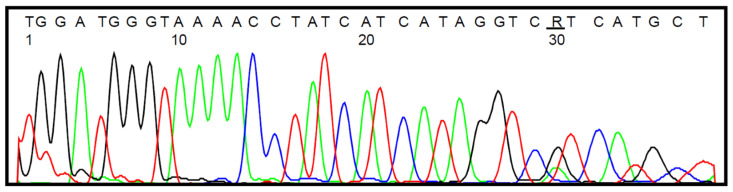
DNA sequencing data for *IDH1* target amplicons showing the heterozygous *IDH1^R132H/WT^* mutation in tumor sample #24 derived from GBM patient (the nucleotide of interest is underlined); SNP accession number: rs121913500.

Selected samples were also subjected to bisulfite sequencing, which in all cases confirmed the data obtained by the HRM analysis. The representative histograms are presented in Figure 2 (wild-type *IDH1* status) and Figure 3 (heterozygous *IDH1^R132H/WT^* mutation).

### 3.3. ABCB1 Methylation Is a Hallmark of Higher Grade Gliomas

**Figure 4:**
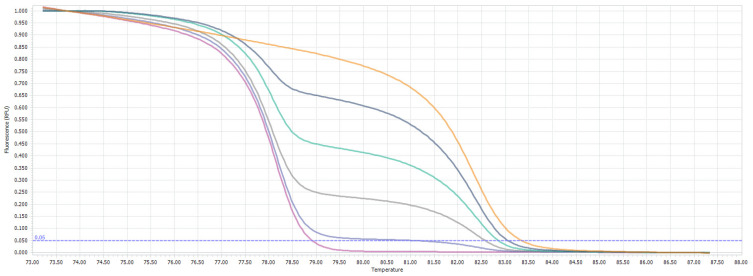
MS-HRM profile of *ABCB1* promoter. Yellow melting curve—100% methylated standard; dark grey—75% methylated, green—50% methylated, light grey—25% methylated, blue—10% methylated, purple—unmethylated standard. For clarity, one replicate of each analyzed sample/control is shown.

**Figure 5:**
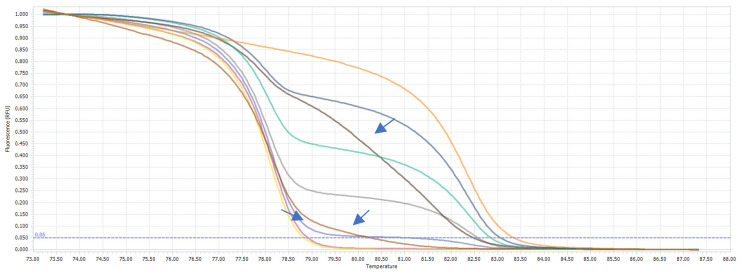
MS-HRM profile of *ABCB1* promoter with representative samples (indicated by the arrows) of glioma patients: Methylated sample derived from a GBM patient #15 (dark brown melting curve across 25–75% methylated standards), mildly methylated samples derived from anaplastic astrocytoma patient #31 (light brown melting curve crossing the 10% methylated standard), and unmethylated sample derived from GBM patient #24 (light yellow sample following the unmethylated standard curve). Yellow melting curve—100% methylated standard; dark grey—75% methylated, green—50% methylated, light grey—25% methylated, blue—10% methylated, purple—unmethylated standard.

The methylation analysis revealed that ABCB1 is frequently methylated in the glioma samples (Table 2). Out of the 50 glioma samples analyzed, ABCB1 was found methylated (at a methylation level higher than 10%) in 52% of the samples (26/50 samples); a mild methylation of ABCB1 (methylation level equals 10%) was detected in 14.0% of the samples (7/50), while unmethylated ABCB1 was found in 34.0% of the samples (17/50). Human astrocyte DNA was also found to be unmethylated. The MS-HRM profile of the standard solutions is presented in Figure 4, while the representative glioma samples together with the standards are shown in Figure 5.

**Figure 6:**
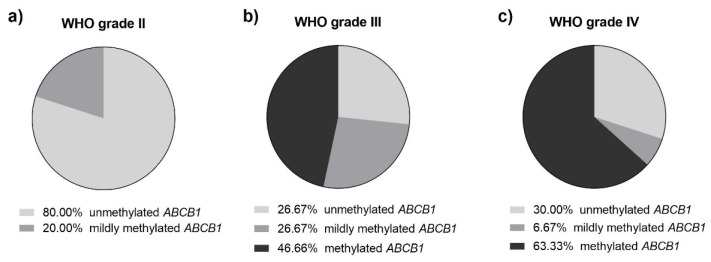
Pie charts presenting the percentage of tumor samples with unmethylated, mildly methylated, or methylated *ABCB1* promoter among patients with (**a**) WHO grade II, (**b**) WHO grade III, and (**c**) WHO grade IV gliomas.

Importantly, the results of the Fisher–Freeman–Halton test revealed that *ABCB1* was more frequently methylated in grade IV gliomas compared to grades II and III (*p* = 0.02514). As presented in Figure 6, among the WHO grade IV gliomas, the majority of the samples (63.33%) had methylated *ABCB1*, while in the WHO grade III it was less than half of the samples (46.66%); in the WHO grade II gliomas, none of the samples were found to be methylated in the *ABCB1* promoter region.

**Figure 7:**
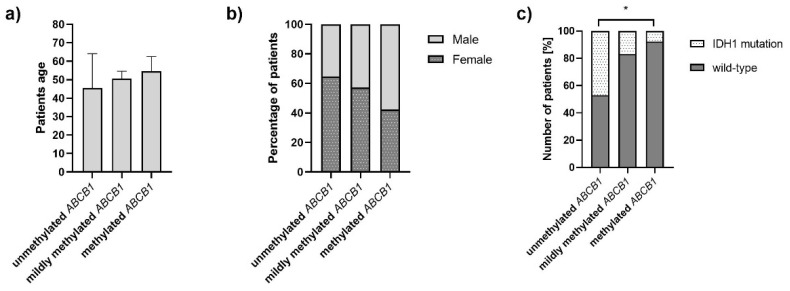
Bar charts presenting the distribution of *ABCB1* methylation among glioma patients of (**a**) different age, (**b**) sex, and (**c**) *IDH1* mutation status. Statistically significant difference is indicated with the asterics (*).

Moreover, as presented in Figure 7a,b, respectively, *ABCB1* methylation was not associated with the patients’ age (*p* = 0.2066) nor did it correlate with patients’ sex (*p* = 0.3365). However, *ABCB1* methylation was significantly more frequent among patients with *IDH1* wild-type status as compared to those with a heterozygous *IDH1^R132H/WT^* mutation (*p* = 0.00963) (Figure 7c). On the other hand, unmethylated *ABCB1* was detected irrespective of the *IDH1* mutation status.

## 4. Discussion

The role of ABC transporters in the MDR of glioma cells has been evaluated over the last few decades [7]. As a result, many efforts have been made to modulate these transporters in order to increase the intracellular concentration of drugs and reverse MDR. Currently, novel synthetic compounds and phytochemicals are being evaluated as ABC transporter inhibitors, since many first and second-generation drug candidates did not show satisfying therapeutic effects. They were either too toxic or showed low inhibitory effects [29]. Additionally, novel ABC transporter-based biomarkers are being constantly sought after and tested. So far, mostly genetic changes in the *ABCB1* gene sequence have been reported to be related to gene expression and treatment responses [14]. The subject of epigenetic changes that are characteristic of *ABCB1*, including aberrant promoter methylation, is far less understood in glioma research. Only single studies have analyzed the DNA promoter methylation of *ABCB1* in GBM patients, and the data concerning other tumors of a glial origin, including astrocytoma or oligodendroglioma, are scarce [25].

Thus, the aim of this study was to analyze the prevalence of *ABCB1* methylation in gliomas of different grades and to verify the potential role of this epigenetic modification in glioma patients’ stratification. In order to do so, we used the MS-HRM technique, which is regarded as one of the best methods for DNA methylation analysis of the clinical samples [30,31]. MS-HRM provides semi-quantitative information on the average methylation status across the CpGs in the target region. The results can be further dichotomized into methylated or unmethylated types based on the chosen threshold. In this study, we trichotomized the results such that 0% methylation was regarded as unmethylated *ABCB1*, 10% as mildly methylated *ABCB1,* and >10% methylation as methylated *ABCB1*. Such a distinction can be very useful in a clinical setting.

The results of this study indicate a high interindividual variability of *ABCB1* methylation between glioma patients, which ranged from 0% to more than 50%. Similar findings were reported by Oberstadt et al., who found high interindividual variability in the promoter methylation status of *ABCB1* among GBM patients. The methylation level of *ABCB1* determined by pyrosequencing was found to vary between 1.3–85.4% [25].

We also found that *ABCB1* methylation is a frequent phenomenon in glioma samples; out of the 50 glioma samples analyzed, *ABCB1* was found methylated in more than half of the samples. More importantly, we also observed a significant correlation between *ABCB1* methylation and a higher WHO grade of glioma. Thus, *ABCB1* methylation can be regarded as a potential biomarker of glioma’s aggressiveness. To the best of our knowledge, this finding has never been reported before.

The 2016 WHO classification divided GBMs according to *IDH1/2* gene status into wild type and mutant GBMs. Importantly, herein, we also found a significant correlation between *ABCB1* methylation and the wild-type status of the *IDH1* gene. This is a new finding, shedding light on the relationship between *IDH1* mutation and methylation alterations in gliomas. In this context, Duncan et al. found that *IDH1^R132H/WT^* mutation induces widespread alterations in DNA methylation, including the hypermethylation of the 2010 and hypomethylation of the 842 CpG loci [24]. Many of these alterations were consistent with those observed in *IDH1*-mutant and G-CIMP+ primary gliomas [24]. Moreover, recent insights in metabolomic studies have suggested a key role of wild-type IDH enzymes upon treatment to favor GBM proliferation and recurrence [32]. Our findings are in line with these observations, suggesting that *ABCB1* methylation is more frequent in *IDH1* wild-type patients compared to those with a heterozygous *IDH1*^R132H/WT^ mutation, who have a much better prognosis. In fact, according to the literature, *IDH* mutant GBM patients have a 5-year survival rate of approximately 80% [33].

Moreover, we did not find a correlation of *ABCB1* methylation with the patients’ age, despite the very certain hypothesis that the processes responsible for the malignant transformation of brain tissue may differ between adults from different age groups [4]. This hypothesis was confirmed in our previous studies, where *RUNX3* and *SFRP1* methylation correlated with the patients’ age [34,35]. Thus, the unexpected lack of correlation between *ABCB1* methylation and patients’ age remains to be elucidated in future studies using a larger cohort of patients. We also did not observe any significant difference in the frequency of *ABCB1* methylation detected among men and women. This is in line with other reports, also investigating different than glioma tumor types [6].

In this study, we proposed *ABCB1* methylation as a new valuable DNA methylation-based biomarker to stratify glioma patients according to the aggressiveness of the tumor, and to facilitate an accurate diagnosis and prognosis of gliomas. This potential biomarker could help develop more personalized treatment protocols for glioma patients, and eventually prolong their survival times. Ultimately, as many more clinically relevant biomarkers are yet to be discovered, the research on epigenetic changes, in particular aberrant DNA methylation studies, should be intensified.

## Figures and Tables

**Table 1:** Primer sequences—with their annealing temperature and amplicon size—for MS-HRM of *ABCB1*, and genotyping of *IDH1*. Primer reverse for *IDH1* genotyping was also used as a sequencing primer during Sanger sequencing.

Gene	Primer	Primer Sequence (5′–3′)	Annealing Temp. (°C)	Product Size (bp)
*ABCB1*	pF	AGATTTAGGAGTTTTTGGAGTAG	56	101
	pR	CTCAAAAAACAAATCCCC		
*IDH1*	pF	GGCTTGTGAGTGGATGGGTA	54	90
	pR	GCAAAATCACATTATTGCCAAC		

**Table 2:** Patients’ characteristics and the results from *IDH1* mutation and *ABCB1* methylation analysis.

No.	Histopathological Classification	WHO Grade	Sex	Age [years]	*IDH1*	*ABCB1* Meth.
1	GBM	IV	M	47	Wild type	>10, <25
2	GBM	IV	M	67	Wild type	>25, <75
3	GBM	IV	M	46	Wild type	>10, <75
4	GBM	IV	F	53	Wild type	>10, <50
5	GBM	IV	M	46	Wild type	>10, <25
6	GBM	IV	F	63	Heterozygous	0
7	GBM	IV	F	63	Heterozygous	0
8	GBM	IV	M	60	Wild type	>25, <100
9	GBM	IV	F	56	Wild type	10
10	GBM	IV	F	60	Wild type	>10, <25
11	GBM	IV	F	56	Wild type	>10, <25
12	GBM	IV	F	56	Wild type	>10, <25
13	GBM	IV	F	57	Wild type	>10, <50
14	GBM	IV	M	60	Wild type	>25, <75
15	GBM	IV	F	54	Wild type	>25, <75
16	GBM	IV	F	48	Wild type	>10, <50
17	GBM	IV	F	47	Wild type	>10, <75
18	GBM	IV	M	52	BD	10
19	GBM	IV	F	51	Wild type	>25, <75
20	GBM	IV	F	47	Wild type	0
21	GBM	IV	M	48	Wild type	>10, <50
22	GBM	IV	M	71	Wild type	0
23	GBM	IV	F	22	Wild type	0
24	GBM	IV	F	28	Heterozygous	0
25	GBM	IV	F	62	Wild type	>10, <50
26	GBM	IV	F	62	Wild type	0
27	GBM	IV	M	71	Wild type	>10, <50
28	GBM	IV	F	69	Wild type	0
29	Gliosarcoma	IV	M	75	Wild type	0
30	Anaplastic astrocytoma	III	M	53	Heterozygous	>10, <25
31	Anaplastic astrocytoma	III	F	49	Wild type	10
32	Anaplastic astrocytoma	III	M	48	Wild type	>10, <25
33	Anaplastic astrocytoma	III	M	48	Wild type	>10, <25
34	Anaplastic astrocytoma	III	M	75	Wild type	>10, <50
35	Anaplastic glioma, recurrent	III	M	45	Wild type	10
36	Oligoastrocytoma	III	M	47	Wild type	>10, <25
37	Oligoastrocytoma	III	M	38	Heterozygous	0
38	Oligoastrocytoma	IV	M	55	Wild type	>10, <50
39	Oligoastrocytoma	III	F	55	Wild type	10
40	Oligoastrocytoma	III	M	23	Wild type	0
41	Oligoastrocytoma	II	F	36	Wild type	0
42	Anaplastic oligoastrocytoma	III	F	45	Heterozygous	0
43	Anaplastic oligoastrocytoma	III	F	47	Wild type	10
44	Anaplastic oligodendroglioma, recurrent	III	M	57	Wild type	>10, <50
45	Anaplastic oligodendroglioma, recurrent	III	M	31	Heterozygous	0
46	Oligodendroglioma	III	F	46	Heterozygous	>10, <50
47	Fibrillary astrocytoma	II	M	50	Heterozygous	10
48	Fibrillary astrocytoma	II	M	50	Heterozygous	0
49	Fibrillary astrocytoma	II	F	29	Heterozygous	0
50	Ganglioglioma	II	F	21	Wild type	0

## Data Availability

The datasets supporting the conclusions of this article are included within the article and are stored in the databases of Poznan University of Medical Sciences.

## References

[1] Louis D.N., Perry A., Reifenberger G., von Deimling A., Figarella-Branger D., Cavenee W.K., Ohgaki H., Wiestler O.D., Kleihues P., Ellison D.W. (2016). The 2016 World Health Organization Classification of Tumors of the Central Nervous System: A Summary. Acta Neuropathol..

[2] Torp S.H., Solheim O., Skjulsvik A.J. (2022). The WHO 2021 Classification of Central Nervous System Tumours: A Practical Update on What Neurosurgeons Need to Know-a Minireview. Acta Neurochir..

[3] Louis D.N., Perry A., Wesseling P., Brat D.J., Cree I.A., Figarella-Branger D., Hawkins C., Ng H.K., Pfister S.M., Reifenberger G. (2021). The 2021 WHO Classification of Tumors of the Central Nervous System: A Summary. Neuro-Oncol..

[4] Sun Y., Xiong Z.-Y., Yan P.-F., Jiang L.-L., Nie C.-S., Wang X. (2019). Characteristics and Prognostic Factors of Age-Stratified High-Grade Intracranial Glioma Patients: A Population-Based Analysis. Bosn. J. Basic Med. Sci..

[5] Spiegl-Kreinecker S., Lötsch D., Ghanim B., Pirker C., Mohr T., Laaber M., Weis S., Olschowski A., Webersinke G., Pichler J. (2015). Prognostic Quality of Activating TERT Promoter Mutations in Glioblastoma: Interaction with the Rs2853669 Polymorphism and Patient Age at Diagnosis. Neuro-Oncol..

[6] Zappe K., Cichna-Markl M. (2020). Aberrant DNA Methylation of ABC Transporters in Cancer. Cells.

[7] Ahmed M., Verreault M., Declèves X., Idbaih A. (2021). Role of Multidrug Resistance in Glioblastoma Chemoresistance: Focus on ABC Transporters. Glioblastoma Resistance to Chemotherapy: Molecular Mechanisms and Innovative Reversal Strategies.

[8] Bodor M., Kelly E.J., Ho R.J. (2005). Characterization of the Human MDR1 Gene. AAPS J..

[9] Hodges L.M., Markova S.M., Chinn L.W., Gow J.M., Kroetz D.L., Klein T.E., Altman R.B. (2011). Very Important Pharmacogene Summary: ABCB1 (MDR1, P-Glycoprotein). Pharmacogenet. Genom..

[10] Reed K., Hembruff S.L., Laberge M.L., Villeneuve D.J., Côté G.B., Parissenti A.M. (2008). Hypermethylation of the ABCB1 Downstream Gene Promoter Accompanies ABCB1 Gene Amplification and Increased Expression in Docetaxel-Resistant MCF-7 Breast Tumor Cells. Epigenetics.

[11] Haenisch S., Zimmermann U., Dazert E., Wruck C.J., Dazert P., Siegmund W., Siegmund S., Kroemer H.K., Warzok R.W., Cascorbi I. (2007). Influence of Polymorphisms of ABCB1 and ABCC2 on MRNA and Protein Expression in Normal and Cancerous Kidney Cortex. Pharm. J..

[12] Ieiri I. (2012). Functional Significance of Genetic Polymorphisms in P-Glycoprotein (MDR1, ABCB1) and Breast Cancer Resistance Protein (BCRP, ABCG2). Drug Metab. Pharmacokinet..

[13] Schaich M., Kestel L., Pfirrmann M., Robel K., Illmer T., Kramer M., Dill C., Ehninger G., Schackert G., Krex D. (2009). A MDR1 (ABCB1) Gene Single Nucleotide Polymorphism Predicts Outcome of Temozolomide Treatment in Glioblastoma Patients. Ann. Oncol. Off. J. Eur. Soc. Med. Oncol..

[14] Malmström A., Łysiak M., Åkesson L., Jakobsen I., Mudaisi M., Milos P., Hallbeck M., Fomichov V., Broholm H., Grunnet K. (2020). ABCB1 Single-Nucleotide Variants and Survival in Patients with Glioblastoma Treated with Radiotherapy Concomitant with Temozolomide. Pharm. J..

[15] Henrique R., Oliveira A.I., Costa V.L., Baptista T., Martins A.T., Morais A., Oliveira J., Jerónimo C. (2013). Epigenetic Regulation of MDR1 Gene through Post-Translational Histone Modifications in Prostate Cancer. BMC Genom..

[16] Vaclavikova R., Klajic J., Brynychova V., Elsnerova K., Alnaes G.I.G., Tost J., Kristensen V.N., Rob L., Kodet R., Skapa P. (2019). Development of High-resolution Melting Analysis for ABCB1 Promoter Methylation: Clinical Consequences in Breast and Ovarian Carcinoma. Oncol. Rep..

[17] Li A., Song J., Lai Q., Liu B., Wang H., Xu Y., Feng X., Sun X., Du Z. (2016). Hypermethylation of ATP-Binding Cassette B1 (ABCB1) Multidrug Resistance 1 (MDR1) Is Associated with Cisplatin Resistance in the A549 Lung Adenocarcinoma Cell Line. Int. J. Exp. Pathol..

[18] Nakai E., Park K., Yawata T., Chihara T., Kumazawa A., Nakabayashi H., Shimizu K. (2009). Enhanced MDR1 Expression and Chemoresistance of Cancer Stem Cells Derived from Glioblastoma. Cancer Invest..

[19] De Gooijer M.C., de Vries N.A., Buckle T., Buil L.C.M., Beijnen J.H., Boogerd W., van Tellingen O. (2018). Improved Brain Penetration and Antitumor Efficacy of Temozolomide by Inhibition of ABCB1 and ABCG2. Neoplasia.

[20] Goldwirt L., Beccaria K., Carpentier A., Farinotti R., Fernandez C. (2014). Irinotecan and Temozolomide Brain Distribution: A Focus on ABCB1. Cancer Chemother. Pharmacol..

[21] Zhang Y., Wang S.-X., Ma J.-W., Li H.-Y., Ye J.-C., Xie S.-M., Du B., Zhong X.-Y. (2015). EGCG Inhibits Properties of Glioma Stem-like Cells and Synergizes with Temozolomide through Downregulation of P-Glycoprotein Inhibition. J. Neurooncol..

[22] Riganti C., Salaroglio I.C., Caldera V., Campia I., Kopecka J., Mellai M., Annovazzi L., Bosia A., Ghigo D., Schiffer D. (2013). Temozolomide Downregulates P-Glycoprotein Expression in Glioblastoma Stem Cells by Interfering with the Wnt3a/Glycogen Synthase-3 Kinase/β-Catenin Pathway. Neuro-Oncol..

[23] Majidinia M., Mirza-Aghazadeh-Attari M., Rahimi M., Mihanfar A., Karimian A., Safa A., Yousefi B. (2020). Overcoming Multidrug Resistance in Cancer: Recent Progress in Nanotechnology and New Horizons. IUBMB Life.

[24] Duncan C.G., Barwick B.G., Jin G., Rago C., Kapoor-Vazirani P., Powell D.R., Chi J.-T., Bigner D.D., Vertino P.M., Yan H.A. (2012). Heterozygous IDH1R132H/WT Mutation Induces Genome-Wide Alterations in DNA Methylation. Genome Res..

[25] Oberstadt M.C., Bien-Möller S., Weitmann K., Herzog S., Hentschel K., Rimmbach C., Vogelgesang S., Balz E., Fink M., Michael H. (2013). Epigenetic Modulation of the Drug Resistance Genes MGMT, ABCB1 and ABCG2 in Glioblastoma Multiforme. BMC Cancer.

[26] Nakayama M., Wada M., Harada T., Nagayama J., Kusaba H., Ohshima K., Kozuru M., Komatsu H., Ueda R., Kuwano M. (1998). Hypomethylation Status of CpG Sites at the Promoter Region and Overexpression of the Human MDR1 Gene in Acute Myeloid Leukemias. Blood.

[27] Nakano H., Nakamura Y., Soda H., Kamikatahira M., Uchida K., Takasu M., Kitazaki T., Yamaguchi H., Nakatomi K., Yanagihara K. (2008). Methylation Status of Breast Cancer Resistance Protein Detected by Methylation-Specific Polymerase Chain Reaction Analysis Is Correlated Inversely with Its Expression in Drug-Resistant Lung Cancer Cells. Cancer.

[28] Yokogami K., Yamasaki K., Matsumoto F., Yamashita S., Saito K., Tacheva A., Mizuguchi A., Watanabe T., Ohta H., Takeshima H. (2018). Impact of PCR-Based Molecular Analysis in Daily Diagnosis for the Patient with Gliomas. Brain Tumor Pathol..

[29] Xiao H., Zheng Y., Ma L., Tian L., Sun Q. (2021). Clinically-Relevant ABC Transporter for Anti-Cancer Drug Resistance. Front. Pharmacol..

[30] Majchrzak-Celińska A., Dybska E., Barciszewska A.-M. (2020). DNA Methylation Analysis with Methylation-Sensitive High-Resolution Melting (MS-HRM) Reveals Gene Panel for Glioma Characteristics. CNS Neurosci. Ther..

[31] Amornpisutt R., Sriraksa R., Limpaiboon T. (2012). Validation of Methylation-Sensitive High Resolution Melting for the Detection of DNA Methylation in Cholangiocarcinoma. Clin. Biochem..

[32] Alzial G., Renoult O., Paris F., Gratas C., Clavreul A., Pecqueur C. (2022). Wild-Type Isocitrate Dehydrogenase under the Spotlight in Glioblastoma. Oncogene.

[33] Cosnarovici M.M., Cosnarovici R.V., Piciu D. (2021). Updates on the 2016 World Health Organization Classification of Pediatric Tumors of the Central Nervous System—A Systematic Review. Med. Pharm. Rep..

[34] Majchrzak-Celińska A., Paluszczak J., Szalata M., Barciszewska A.-M., Nowak S., Kleszcz R., Sherba A., Baer-Dubowska W. (2015). The Methylation of a Panel of Genes Differentiates Low-Grade from High-Grade Gliomas. Tumour Biol. J. Int. Soc. Oncodev. Biol. Med..

[35] Majchrzak-Celińska A., Słocińska M., Barciszewska A.-M., Nowak S., Baer-Dubowska W. (2016). Wnt Pathway Antagonists, SFRP1, SFRP2, SOX17, and PPP2R2B, Are Methylated in Gliomas and SFRP1 Methylation Predicts Shorter Survival. J. Appl. Genet..

